# Association between chronic kidney disease and the most common corneal ectasia disease (keratoconus): a nationwide cohort study

**DOI:** 10.1186/s12882-021-02307-z

**Published:** 2021-03-25

**Authors:** Ren-Long Jan, Shih-Feng Weng, Jhi-Joung Wang, Yuh-Shin Chang

**Affiliations:** 1 Graduate Institute of Medical Science, College of Health Science, Chang Jung Christian University, Tainan, Taiwan; 2 Department of Pediatrics, Chi Mei Medical Center, Liouying, Tainan, Taiwan; 3 Department of Healthcare Administration and Medical Informatics, Kaohsiung Medical University, Kaohsiung, Taiwan; 4 Department of Medical Research, Kaohsiung Medical University Hospital, Kaohsiung, Taiwan; 5 Center for Medical Informatics and Statistics, Office of R&D, Kaohsiung Medical University, Kaohsiung, Taiwan; 6 Department of Medical Research, Chi Mei Medical Center, Tainan, Taiwan; 7 Department of Anesthesiology, Chi Mei Medical Center, Tainan, Taiwan; 8 AI Biomed Center, Southern Taiwan University of Science and Technology, Tainan, Taiwan; 9 Department of Ophthalmology, Chi Mei Medical Center, Tainan, Taiwan

**Keywords:** Chronic kidney disease, Keratoconus, Taiwan Longitudinal Health Insurance Database, Cohort study

## Abstract

**Background:**

Both keratoconus (KCN) and chronic kidney disease (CKD) are multifactorial conditions with multiple aetiologies and share several common pathophysiologies. However, the few studies that have described the relationship between KCN and CKD are limited to case reports and small case series. This study aimed to evaluate the association between KCN and CKD.

**Methods:**

The study cohort included 4,609 new-onset keratoconus patients ≥ 12 years identified by the International Classification of Diseases, Ninth Revision, Clinical Modification, code 371.6 and recruited between 2004 and 2011 from the Taiwan National Health Insurance Research Database. The age-, sex-, and comorbidity-matched control group included 27,654 non-KCN patients, selected from the Taiwan Longitudinal Health Insurance Database, 2000. Information for each patient was collected and tracked from the index date until December 2013. The incidence and risk of CKD were compared between the two groups. The adjusted hazard ratios (HRs) for CKD were calculated with Cox proportional hazard regression analysis. Kaplan–Meier analysis was used to calculate the cumulative CKD incidence rate.

**Results:**

The incidence rate of CKD was 1.36 times higher in KCN patients than in controls without statistically significant difference (95 % confidence interval [CI] = 0.99–1.86, *p* = 0.06). In total, 29 male KCN patients and 90 male controls developed CKD during the follow-up period. The incidence rate of CKD was 1.92 times (95 % [CI] = 1.26–2.91; *p* = 0.002) higher in male KCN patients than in controls. After adjusting for potential confounders, including age, hypertension, hyperlipidaemia, and diabetes mellitus, male KCN patients were 1.75 times (adjusted HR = 1.75, 95 % [CI] = 1.14–2.68, *p* < 0.05) more likely to develop CKD.

**Conclusions:**

We found that male KCN patients have an increased risk of CKD. Therefore, it is recommended that male KCN patients should be aware of CKD.

## Background

Keratoconus (KCN), the most common ectatic disease of the cornea, is a bilateral, asymmetric, and progressive ectatic condition. The disease is characterized by progressive thinning and steepening of the cornea, resulting in a conical shaped cornea and significant visual impairment [1]. The disease has significant visual morbidity and eventually 15–20 % of patients with KCN will require a corneal transplant making it the leading cause of keratoplasty in the developed world [2]. KCN affects all ethnicities and both genders. It typically presents in adolescence with a 10–20 year progression until it reaches a stable phase in the third or fourth decade [3]. Despite being the most common of the ectatic diseases of the cornea, the incidence of KCN is low (2.38 cases per 100,000 person-years) in Taiwan [4] and its aetiology and pathophysiology are not fully understood. The cellular aetiology of the disease has been evaluated genetically, biochemically, and physically, and it has been suggested that the disease may be multifactorial in origin [5].

Chronic kidney disease (CKD) has become a major public health issue due to its increasing prevalence worldwide in recent years [6]. CKD is defined by a reduction in the estimated glomerular filtration rate (eGFR) or elevated urinary albumin, or both [7, 8]. CKD is marked by a progressive and persistent damage or loss of kidney parenchyma. This irreversible reduction in the number of functioning nephrons results in a gradual decline of renal maintenance of homeostasis and a progressive loss in renal function over a period of weeks to years eventually progressing to end-stage renal disease [9]. The major pathological changes seen in CKD such as glomerulosclerosis and renal interstitial fibrosis [10], are associated with extracellular matrix (ECM) remodelling due to dysregulation of proteases [11, 12], and inflammatory process [13].

Both KCN and CKD are multifactorial conditions with multiple aetiologies and pathophysiologies. The corneal stroma and kidney mesangial matrix are rich in collagen components [12, 14]. The dysregulation of collagen components by a variety of proteases, such as matrix metalloproteinases (MMP), leading to ECM remodelling plays a key role in the development of these diseases [11, 12, 15, 16]. Additionally, although KCN is regarded as a non-inflammatory condition, several studies found high inflammatory molecules including interleukin-6 (IL-6) and tumour necrosis factor (TNF-α) in the tears of KCN patients and implied that there may be an inflammatory component in the development of KCN [16–18]. It is worth noting that inflammation due to increased proinflammatory cytokines and other inflammatory factors has also been implicated in the pathogenesis of CKD [13, 19]. Finally, recent studies have highlighted the effects of sex hormones on the corneas of KCN patients [20, 21]. It has been postulated that the progression of CKD is linked to an increase in renal fibrosis resulting from the activation of the mineralocorticoid receptor (MR) related to aldosterone [22–24]. Based on these common pathogenic mechanisms, it is clinically relevant to examine KCN as a possible risk factor for CKD.

Previous studies have discussed the association between KCN and renal disease [25–27], but their results were limited to case reports and small case series. Therefore, we designed this study to clarify the association between KCN and CKD, using a nationwide population-based data set.

## Methods

### Database

The data used in our study is from the National Health Insurance Research Database (NHIRD), which is provided by the National Health Research Institute (NHRI). The NHIRD records each beneficiary’s coded information regarding patient birth date, sex, and residential area, as well as the International Classification of Diseases, Ninth Revision, Clinical Modification (ICD-9-CM) diagnoses and prescriptions details, procedures, and expenditure, regardless of whether the patient is under ambulatory care or during hospitalization. 

### Selection of patients and variables

**Fig. 1:**
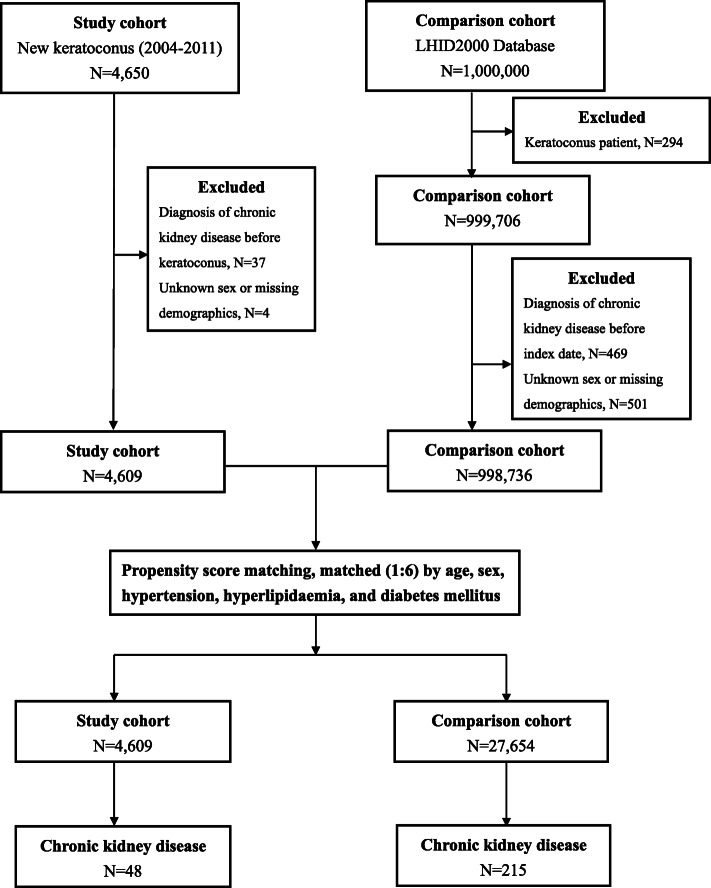
Flowchart demonstrating the enrollment process for patients with keratoconus and the controls

Two groups including a new-onset KCN group and a matched non-KCN control group were enrolled in this retrospective cohort study. We collected patient information from both groups from the beginning of 2004 to the end of 2011 and traced both groups until December 31, 2013. A flowchart of our study is shown in Fig. 1. Initially, 4,650 patients with a diagnosis of KCN (ICD-9-CM code 371.6) ≥ 12 years were included. We excluded 4 patients with unknown sex or missing demographic data, as well as 37 patients diagnosed with CKD (ICD-9-CM codes 585, 586, 403.11, 403.91, 404.02, 404.03, 404.12, 404.13, 404.92, and 404.93) before KCN. Finally, there were 4,609 KCN patients ≥ 12 years in the KCN group.

For each KCN patient, we randomly selected six non-KCN controls from the longitudinal Health Insurance Database 2000 (LHID 2000), which is a subset of the NHIRD, containing overall claim data from one million beneficiaries in year 2000 and constructed from the NHIRD by the NHRI using a systematic sampling method. From the one million beneficiaries in the LHID 2000, we excluded 294 who had been previously diagnosed with KCN, and further excluded 501 patients with unknown sex or missing demographic data and 469 patients diagnosed with CKD before the index date. Controls (*n* = 998,736) were matched with KCN patients via propensity scores by age, sex, comorbidities including hypertension, hyperlipidaemia, and diabetes mellitus. The index date for KCN patients was defined as the date of diagnosis of KCN, and the index date for the control patients was assigned based on the KCN subject’s index date after matching by year of birth. This implies that the index date was the same date in the KCN and control groups for each pair. Afterward, controls were matched with KCN patients via propensity scores by age; sex; comorbidities including hypertension, hyperlipidemia, and diabetes mellitus. The propensity score matching was used to reduce selection bias, because it enables grouping of the numerous confounding covariates in an observational study, that has a large number of variables. An SAS (SAS Institute, USA) matching macro, “%OneToManyMTCH,” was used for this matching. It allows propensity score matching from 1-to-1 to 1-to-N. We set a calliper for nearest-neighbour matching within the first four to eight digits; for example, two patients with propensity scores of 0.12345678 and 0.12347123 are matched on the first two digits (0.12). The macro makes the “best” matches first and the “next-best” matches next in a hierarchical sequence until no more matches can be made. We tracked each participant in both groups from the index date until December 31, 2013 or death, whichever was earlier. Demographic data of the participants in both groups were recorded. Additionally, data regarding their comorbidities including hypertension (ICD-9-CM codes 401–405), hyperlipidaemia (ICD-9-CM code 272), and diabetes mellitus (ICD-9-CM code 250) were collected, because these are risk factors of CKD. We included the comorbidities only if the condition occurred in an inpatient setting or if they appeared in 3 or more ambulatory care claims within 1 year before the index date.

### Statistical analysis

All statistical analyses were performed using SAS 9.4 for Windows (SAS Institute, Inc., Cary, NC, USA). The Pearson’s chi-square test was used to compare baseline demographics and comorbidities between the KCN and controls. The median follow-up time for the KCN and the control groups was compared by Wilcoxon rank sum test. The CKD incidence was measured as the sum of KCN patients detected during the follow-up period divided by the total person-years (PY) for each group by age, sex, and selected comorbidities. We performed Poisson regression analysis to calculate the incidence rate ratio (IRR), which compared the risk of CKD between the KCN and non-KCN control groups. Cox proportional hazards regression analysis was utilized to calculate the difference in the adjusted hazard ratios (HRs) and 95 % confidence intervals (CIs) to determine the risk of developing CKD. We also examine the statistical the interactions KCN*age and KCN*Hypertension. Kaplan–Meier analyses and log rank tests were used to obtain the cumulative incidence rates and analyse the differences, respectively. Statistical significance was defined as *p* < 0.05.

## Results

### Demographic data

After excluding ineligible subjects, 4,609 KCN patients and 27,654 controls were recruited in this study from the beginning of 2004 to the end of 2011. The baseline demographics and comorbidities of KCN patients and non-KCN controls are shown in the Table 1. The average age of KCN patients and controls was 27.43 (standard deviation [SD], 13.04) and 27.57 (SD, 12.91) years, respectively. Of the 4,609 KCN patients, 2,508 (54.42 %) were men and 2,101 (45.58 %) were women; 1,271 (27.58 %) were aged 12–19 years, 1,884 (40.88 %) were 20–29 years, 898 (19.48 %) were 30–39 years, and 556 (12.06 %) ≥ 40 years. Regarding comorbidities of the 4,609 KCN patients, 148 (3.21 %) have hypertension, 71 (1.54 %) have hyperlipidaemia, and 63 (1.37 %) have diabetes mellitus. The median follow-up periods for the KCN and control patients were 5.23 years (inter-quartile range 3.52 − 7.46 years) and 5.34 years (inter-quartile range 3.55 − 7.54 years), respectively.

**Table 1:** Demographic characteristics and comorbid disorders between the keratoconus group and controls

	Keratoconus (*N* = 4609)	Controls (*N* = 27,654)	*p*-value
Age (years), mean ± SD	27.43 ± 13.04	27.57 ± 12.91	0.50
Age group	n (%)	n (%)	
12 − 19 years	1271 (27.58)	7563 (23.44)	0.97
20 − 29 years	1884 (40.88)	11,377 (41.14)	
30 − 39 years	898 (19.48)	5340 (19.31)	
≥ 40 years	556 (12.06)	3374 (12.20)	
Gender			
Male	2508 (54.42)	14,804 (53.53)	0.27
Female	2101 (45.58)	12,850 (46.47)	
Baseline comorbidity			
Hypertension	148 (3.21)	868 (3.14)	0.79
Hyperlipidaemia	71 (1.54)	473 (1.71)	0.41
Diabetes mellitus	63 (1.37)	338 (1.22)	0.41
Follow − up time (years)			
Median (IQR)	5.23 (3.52 − 7.46)	5.34 (3.55 − 7.54)	0.08

Note: The demographic characteristics and comorbid disorders between the keratoconus and control groups were compared using Pearson chi-square tests. The median of follow-up time was calculated with Wilcoxon rank sum test

Abbreviations: *IQR* inter-quartile range, *SD* standard deviation

### Incidence rates for CKD

During the follow-up period, the incidence rate of CKD was higher in patients with KCN (18.83/10,000 PY) than in age-matched controls (13.89/10,000 PY) leading to a 1.36 times difference in CKD IRR (1.36, 95 % CI = 0.99–1.86, *p* = 0.06; Table 2) between the two groups. However, this difference was not statistically significant.

**Table 2:** Risk of chronic kidney disease in the keratoconus group and control group

Characteristics	Keratoconus				Controls				IRR (95 % CI)	*p*-value
	N	CKD	PY	Rate^a^	N	CKD	PY	Rate^a^		
All	4609	48	25,491	18.83	27,654	215	154,786	13.89	1.36 (0.99 − 1.86)	0.06
Age (years)										
12 − 19	1271	3	7058	4.25	7563	9	43,593	2.06	2.06 (0.56 − 7.60)	0.28
20 − 29	1884	10	10,742	9.31	11,377	34	64,806	5.25	1.77 (0.88 − 3.59)	0.11
30 − 39	898	7	4717	14.84	5340	24	28,102	8.54	1.74 (0.75 − 4.03)	0.20
≥ 40	556	28	2974	94.15	3374	148	18,284	80.95	1.16 (0.78 − 1.74)	0.46
Gender										
Male	2508	29	14,050	20.64	14,804	90	83,605	10.76	1.92 (1.26 − 2.91)	0.002
Female	2101	19	11,440	16.61	12,850	125	71,180	17.56	0.95 (0.58 − 1.53)	0.82
Comorbidity										
Hypertension	148	15	778	192.80	868	101	4385	230.33	0.84 (0.49 − 1.44)	0.52
Hyperlipidaemia	71	6	323	185.76	473	41	2260	181.42	1.03 (0.44 − 2.41)	0.95
Diabetes mellitus	63	9	322	279.50	338	51	1618	315.20	0.89 (0.44 − 1.80)	0.74

Note: The Poisson regression analysis was performed to calculate the incidence rate ratio

Abbreviations: *CI* confidence interval, *CKD* chronic kidney disease, *IRR* incidence rate ratio, *PY* person-years

^a^Rate: per 10,000 person-years

The incidence rate of CKD was higher in male KCN patients (20.64/10,000 PY) than in the male controls (10.76/10,000 PY). Additionally, there was a significant difference in the IRR of CKD (1.92, 95 % CI = 1.26–2.91, *p* = 0.002; Table 2) between men with KCN and male controls. The IRR for CKD in women with KCN indicated that the risk of CKD was not significantly greater than that in the corresponding controls (Table 2).

Concerning the four age groups, KCN patients aged ≥ 40 years exhibited the highest incidence of CKD (94.15/10,000 PY), followed by those aged 30–39 years (14.84/10,000 PY), 20–29 years (9.31/10,000 PY) and 12–19 years (4.25/10,000 PY). However, there were no significant differences in CKD incidence rates between these age groups and their controls (Table 2).

In the KCN group, the CKD incidence rates decreased in the following order: patients with diabetes mellitus (279.50/10,000 PY), hypertension (192.80/10,000 PY), and hyperlipidaemia (185.76/10,000 PY). The IRR for CKD in KCN patients with these comorbidities indicated that the risk of CKD was not significantly greater in the KCN group than in the corresponding controls (Table 2).

**Fig. 2:**
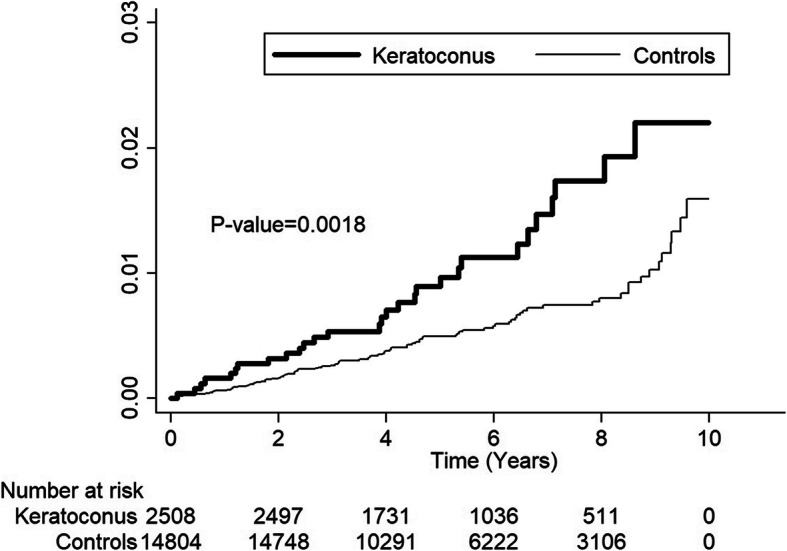
Cumulative incidence of chronic kidney disease in male keratoconus patients and controls during the follow-up period

Kaplan–Meier analyses indicated higher cumulative incidence rates for CKD in males in the KCN group than in non-KCN controls, and log-rank test findings were also significant (*p* = 0.002; Fig. 2).

### Hazard ratios for CKD

Table 3 shows the crude and adjusted HRs for CKD in male participants during the follow-up period. After adjusting for age, and the selected comorbidities, KCN was identified as a risk of CKD for male participants (adjusted HR = 1.75, 95 % CI = 1.14–2.68, *p* < 0.05) despite a strong effect of age and hypertension. In addition, CKD risk increased with age and was higher in patients with hypertension. The interactions KCN*age and KCN*Hypertension were not statistically significant.

**Table 3:** Crude and adjusted hazard ratios of Cox proportional hazard regressions and 95 % confidence interval for chronic kidney disease in males during the follow-up period

Cohort	Crude Hazard Ratio (95 % CI)	Adjusted Hazard Ratio (95 % CI)
Keratoconus		
Yes	1.92 (1.27 − 2.92)*	1.75 (1.14 − 2.68)*
No	1.00	1.00
Age (years)		
12 − 19	1.00	1.00
20 − 29	2.71 (1.18 − 6.22)*	2.67 (1.16 − 6.12)*
30 − 39	5.46 (2.26 − 13.17)*	5.15 (2.13 − 12.44)*
≥ 40	36.92 (16.96 − 80.40)*	19.83 (8.75 − 44.97)*
Comorbidity		
Hypertension		
Yes	24.01 (16.40 − 35.16)*	4.36 (2.59 − 7.32)*
No	1.00	1.00
Hyperlipidaemia		
Yes	10.65 (5.98 − 18.96)*	1.15 (0.61 − 2.19)
No	1.00	1.00
Diabetes mellitus		
Yes	23.20 (14.20 − 37.92)*	1.65 (0.90 − 3.00)
No	1.00	1.00

Note: The adjusted hazard ratio for developing chronic kidney disease in males was calculated using the Cox proportional hazard regression analysis

Abbreviation: *CI* confidence interval

^*^p < 0.05

## Discussion

After a thorough review of relevant research, we found that our study is the largest-scale population-based study to explore the relationship between KCN and subsequent CKD. We analysed 4,609 KCN patients ≥ 12 years and 27,654 matched controls. The results indicated a significantly increased risk of CKD in male KCN patients compared with controls.

To our knowledge, several studies have investigated the association between KCN or lenticonus and various renal disorders [25–27]. This association has been reported in case reports or small case series including Noonan’s syndrome [27], Alport’s syndrome [25], and Leber’s congenital amaurosis [26]. Most of these renal disorders are congenital disorders involving multiple organs. These studies were also limited by small sample sizes. However, Alport syndrome and the other conditions are rare and could not have accounted for a significant increase of CKD in patients with KCN. In contrast, our study enrolled KCN patients ≥ 12 years old and is the largest nationwide, population-based cohort study to investigate the risk of CKD following KCN in Taiwan to date.

Structures of the cornea stroma are rich in ECM composed of collagen components [14]. The intraglomerular mesangial cells of the kidney are also embedded in an ECM matrix comprised of collagen IV, V, fibronectin, laminin, and proteoglycans [12]. Stromal degradation and thinning is the most important feature of KCN. This ECM remodelling is due to increased levels of proteases such as MMPs [16]. Gelatinases MMP-2 and MMP-9, which are dominant regulators of ECM formation and breakdown in the glomerulus, cleave basement membrane components, and degrade the ECM structures of kidney tissues [11, 12]. An upregulation of MMP and dysregulation of MMP/ tissue inhibitor of metalloproteinase activity have been reported in matrix remodelling in different stages of CKD and play a role in the progression of CKD [11, 12, 28]. These similar patterns of remodelling in the extra cellular matrices associated with the over-expression of proteolytic enzymes may imply a link between KCN and CKD.

Although KCN was previously characterized as a non-inflammatory condition, several recent studies have suggested that inflammatory processes play a role in the pathogenesis of KCN [29, 30]. Numerous elevated inflammatory markers, such as IL-6 and TNF-α were found in the tears of KCN patients indicating an inflammatory component in the development of KCN [18, 31]. Increased inflammatory markers may likely be the common pathophysiological mechanism of KCN and CKD. Several studies have found higher inflammatory markers, include IL-6 and TNF-α, in CKD patients compared to healthy controls. The amount of serum IL-6 and TNF-α elevated was consistent with eGFR reduction and reached its highest levels in severe CKD patients [13, 19]. This common increase in proinflammatory cytokines may explain the association KCN with subsequent CKD formation.

The observed male predominance in the subsequent development of CKD in KCN patients may be due to aldosterone related MR activation in CKD. The MR is associated with the activation of a variety of pathological processes including remodelling, fibrosis, and inflammation in CKD [32]. Several studies have found that activation of the MR related to aldosterone may have a major impact on the progression of CKD symptoms including elevated blood pressure, proteinuria, and renal fibrosis [22–24]. Further experimental studies have demonstrated that using MR antagonist agents could slow down the progression of CKD [22–24]. Because sex hormones can act through receptors expressed in the ocular tissue, for example, the effects of androgens rely on the androgen receptors present in corneal tissue, they may be associated with various ocular pathologies. Although Clinical information about sex hormones in KCN patients is scarce, recent studies have investigated the role of hormonal changes on corneal structure in KCN patients [20, 21]. Mckay et al. reported a significant increase of salivary dehydroepiandrosterone sulphate (DHEA-S, a common precursor of androgens) in KCN patients of both genders [20]. Rabab et al. reported in their preliminary studies that exogenous DHEA led to significant upregulation of collagen type III and cellular fibronectin suggesting that DHEA may drive human keratotic cornea towards a fibrotic change [21]. A similar fibrotic change related to androgen or miniralocorticoid may be the link between KCN and subsequent CKD development in male patients.

Unlike KCN which usually occurs during puberty followed by 10–20 years of progression until it stabilises in the third to fourth decade of life, CKD is an age-dependent disease. CKD is a progressive disease that results in end-stage renal disease through pathological renal interstitial fibrosis leading to irreversible loss of renal function [9, 10]. In addition, several studies indicate that the demographics of KCN patients vary widely from 51.0 years to 63.6 years worldwide while CKD patients are usually ≥ 40 years of age [7, 33, 34]. Our study shows similar results with highest risk of CKD in patients 40 and older.

Some studies have reported several comorbidities associated with CKD, such as hypertension, diabetes mellitus, and hyperlipidaemia [7, 8, 35]. We assessed the risk of CKD associated with these comorbidities in our study population of male patients 12 years and older and found that only hypertension was an independent risk factor for CKD. This finding is consistent with some previous studies that identified hypertension as a main risk factor for CKD [7, 8, 35]. Hypertension is a growing non-communicable disease and an important leading cause of CKD [7, 35]. Hypertension accelerates the decline of renal function regardless of aetiology [8]. Maintaining blood pressure below 140/90 mmHg, lifestyle modification, and antihypertensive drug therapy should be advised to patients with hypertension to reduce the risk of cardiovascular events [8, 36].

There are several strengths in our study. First, our nationwide population-based study included a large sample of KCN patients resulting in superior statistical power and precision in risk appraisal. Second, patients with visual disturbances visit ophthalmologists and patients with renal problems visit nephrologists leading to reduced misdiagnoses, and selection bias in referral centres. Third, this cohort study was conducted with longitudinal data of up to 10 years, and potential confounding bias was eliminated by adjusting for hypertension, hyperlipidaemia, and diabetes mellitus.

There are several limitations in this study. Because the medical history of each participant in the study can only be tracked back to the year 1996, we cannot confirm whether the controls had a history of KCN before January 1996. Additionally, several important sociodemographic characteristics such as alcohol consumption, tobacco smoking habits, and laboratory data including blood pressure, blood sugar, or eGFR are not available in the claim database of the NHRI. The eGFR not being available in the claim database of the NHRI is a remarkable limitation, because it should be incorporated at the time of diagnosis of keratoconus in the patient group, or at the beginning of follow up in the control group and should considered as a variable for propensity score matching to reduce bias. Finally, incorrect classification is possible as the diagnosis of KCN, CKD, and other comorbidities relied on ICD-9-codes. The severity of KCN could not be identified for the ICD-9-CM code 371.6 in our study.

## Conclusions

In summary, this study found that the risk of CKD was significantly higher in male KCN patients than their controls and remained an independent risk factor after adjusting for age and other confounders in the male cohort. These results suggest that clinicians should inform male KCN patients about CKD.

## Data Availability

All data generated or analysed during this study are included in this published article.
